# Author Correction: Circularized fluorescent nanodiscs for probing protein–lipid interactions

**DOI:** 10.1038/s42003-022-03859-y

**Published:** 2022-09-01

**Authors:** Qian Ren, Shanwen Zhang, Huan Bao

**Affiliations:** Department of Molecular Medicine, UF Scripps Biomedical Research, 130 Scripps Way, Jupiter, 33458 FL USA

**Keywords:** Biophysical chemistry, Biosensors

Correction to: *Communications Biology* 10.1038/s42003-022-03443-4, published online 26 May 2022.

The authors were under the impression that their findings using plasmid pGEX4T-cpx2 and the C21 peptide were reproduced in another Article (Courtney et al, 10.1038/s41594-021-00716-0) using a different plasmid, petSUMO-cpx2 as described in the Methods section of that paper. However, it has come to light the data shown in the Extended Fig.1a-b of Courtney et al. were actually data obtained using pGEX4T-cpx2 and the C21 peptide. Therefore, in the original Supplementary Information for this Article, the legend to Supplementary figure 6 did not mention that the data used to generate the graphs in panels b and c had been published elsewhere. The Supplementary Information file has been replaced with one containing the correct information and citation.

Further, the Acknowledgements should read: We thank Dr. Michael Farzan for access to the DLS instrument. We thank Drs. Naomi Kamasawa and Debby Guerrero-Given from the imaging center at the Max Planck Florida Institute for Neuroscience for assist in EM. We would also like to thank Dr. Franck Duong for the plasmid of pBad33-EIIA^Glc^, Dr. Anjon Audhya for the plasmid pGEX2T-sar1p, and Dr. Edwin Chapman for the plasmids of pGEX4T-syt1 and pGEX4T-cpx2. This work is supported by the NIH Director’s New Innovator Award (DP2GM140920 to H.B.). Work shown in Supplementary Fig. 6b and c was carried out at UW-Madison and supported by Human Frontier Science Program postdoctoral fellowship no. LT000712 (Huan Bao), Pew Charitable Trust grant no. 864K625 (Edwin R. Chapman and Dorit Hanein) and National Institute of Health grant nos. MH061876 and N097362 (Edwin R. Chapman).

This has now been corrected in the HTML and pdf versions of the Article.

## Supplementary information

Supplementary data Corrected

