# Supplementary material for: Circularized fluorescent nanodiscs for probing protein–lipid interactions
Source: Commun Biol. 2022 May 26;5:507. doi: 10.1038/s42003-022-03443-4 (PMC9135701; doi:10.1038/s42003-022-03443-4)
Supplement: Supplementary file 2 — Supplementary Information [file 42003_2022_3443_MOESM2_ESM.pdf]

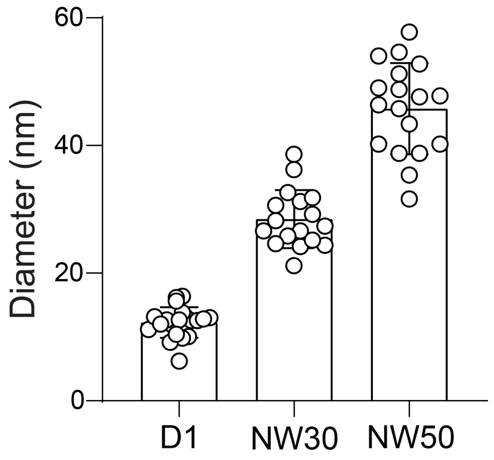


**Supplementary Figure 1. Diameters of split GFP circularized nanodiscs determined by DLS.** **D1**, spGFP$_{1\text{-}10/11}$-MSP1D1; **NW30**, spGFP$_{1\text{-}10/11}$-NW30; **NW50**, spGFP$_{1\text{-}10/11}$-NW50. These measurements showed that the diameters of spGFP$_{1\text{-}10/11}$-MSP1D1, spGFP$_{1\text{-}10/11}$-NW30, and spGFP$_{1\text{-}10/11}$-NW50 nanodiscs were 12, 28, and 46 nm, respectively. Data are shown as mean ± s.d. from multiple measurements using $n \geq 3$ independent sample preparations.

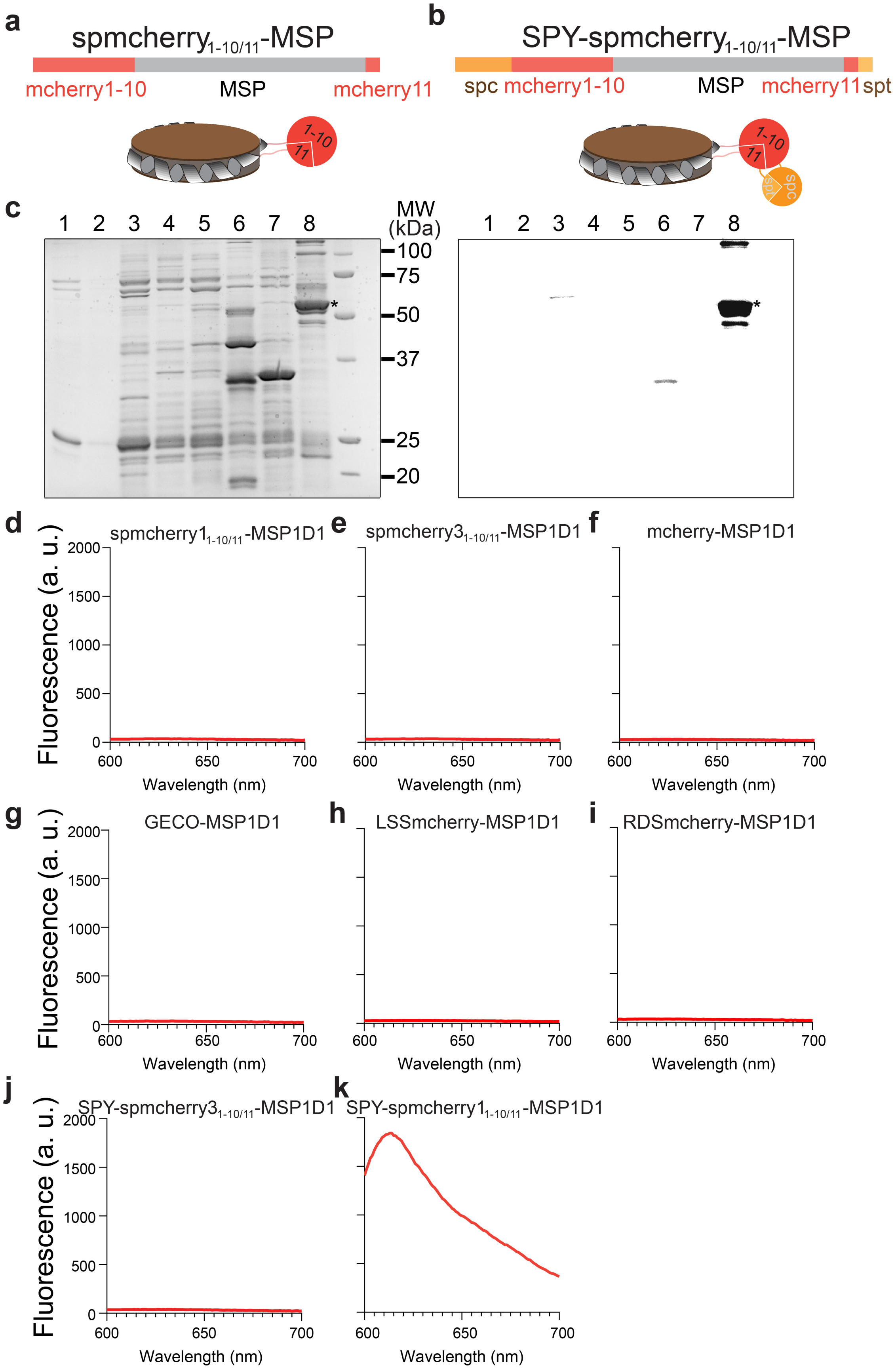

a
spmcherry1-10/11-MSP
mcherry1-10
MSP
mcherry11
b
SPY-spmcherry1-10/11-MSP
spc mcherry1-10
MSP
mcherry11spt
c
1 2 3 4 5 6 7 8
MW (kDa)
100
75
50
37
25
20
1 2 3 4 5 6 7 8
d
spmcherry11-10/11-MSP1D1
e
spmcherry31-10/11-MSP1D1
f
mcherry-MSP1D1
g
GECO-MSP1D1
h
LSSmcherry-MSP1D1
i
RDSmcherry-MSP1D1
j
SPY-spmcherry31-10/11-MSP1D1
k
SPY-spmcherry11-10/11-MSP1D1
Fluorescence (a. u.)
2000
1500
1000
500
0
600
650
700
Wavelength (nm)

**Supplementary Figure 2. Expanding the palette of isenND**.

(**a-b**) Illustration of spmcherry$_{1\text{-}10/11}$-MSP (a) and SPY-spmcherry$_{1\text{-}10/11}$-MSP (b) nanodisc. (**c**) SDS-PAGE of the designed RFP-MSPs pulled down from bacterial cells stained with Coomassie blue (left) and in-gel fluorescence imaging at the mcherry channel (right). **1**, spmcherry1$_{1\text{-}10/11}$-MSP1D1(51 kDa); **2**, spmcherry3$_{1\text{-}10/11}$-MSP1D1(51 kDa); **3**, mcherry-MSP1D1 (51 kDa); **4**, GECO-MSP1D1 (52 kDa); **5**, LSSmcherry-MSP1D1(53 kDa); **6**, RDSmcherry-MSP1D1(53 kDa); **7**, SPY-spmcherry3$_{1\text{-}10/11}$-MSP1D1(65 kDa); **8**, SPY-spmcherry1$_{1\text{-}10/11}$-MSP1D1 (65 kDa). We only found that SPY-spmcherry1$_{1\text{-}10/11}$-MSP1D1 exhibited the expected size and red fluorescence, as indicated by the star (*). (**d-k**) Representative fluorescence emission spectrum of proteins pulled down from cells expressing the indicated scaffold protein. In these trials, we only observed SPY-spmcherry$_{1\text{-}10/11}$-MSP1D1 resulted in the expected typical fluorescence spectrum of RFP.

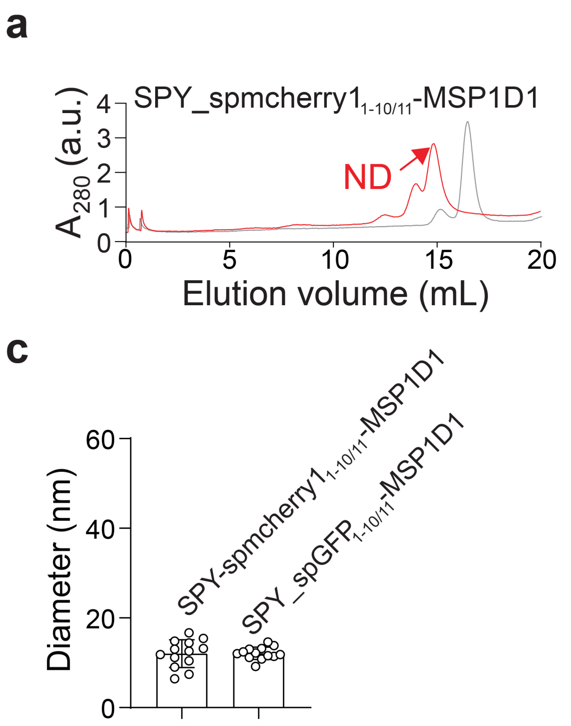


**Supplementary Figure 3. Spycatcher-SpyTag promotes the formation of split GFP and mcherry circularized nanodiscs.** (**a-b**) Representative SEC profiles of the indicated MSP (gray) and nanodisc (red and green). Protein: lipid ratios were 1:60 for nanodisc reconstitution using the indicated MSPs. (**c**) Diameters of purified nanodiscs from SEC (A and B) by DLS measurements. Data are shown as mean ± s.d. from multiple measurements using n ≥ 3 independent sample preparations.

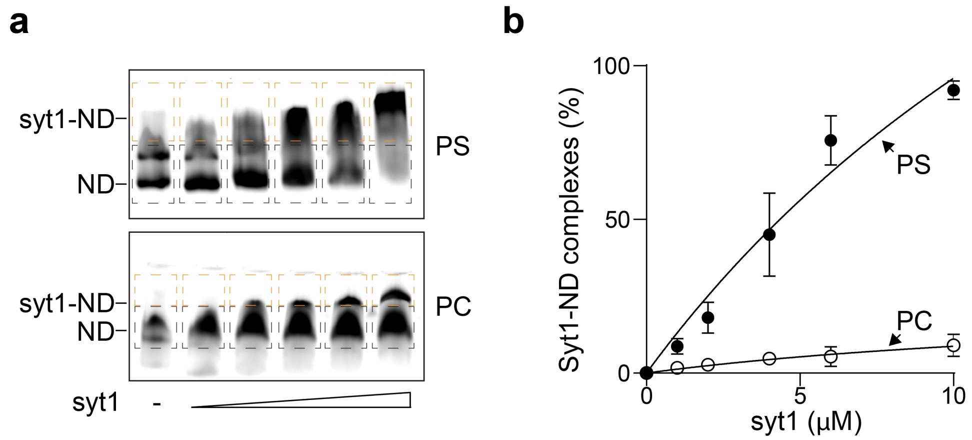


**Supplementary Figure 4. Characterization of syt1 binding to isenND by native PAGE.**
(**a**) Representative native PAGE of syt1 binding to isenND containing PS or PC lipids by in-gel fluorescence imaging at GFP channel. ND (0.2 μM) were incubated with increasing concentrations of syt1 in the presence of $Ca^{2+}$ (0.5 mM) at room temperature for 10 mins. Samples were then subjected to native electrophoresis using Mini-PROTEAN gels from BioRad. The encircled boxes were used for quantification by gel densitometry in panel B. (**b**) Quantification of the syt1-ND complex formation from native PAGE. Data are shown as mean ± s.d., n = 3 independent experiments.

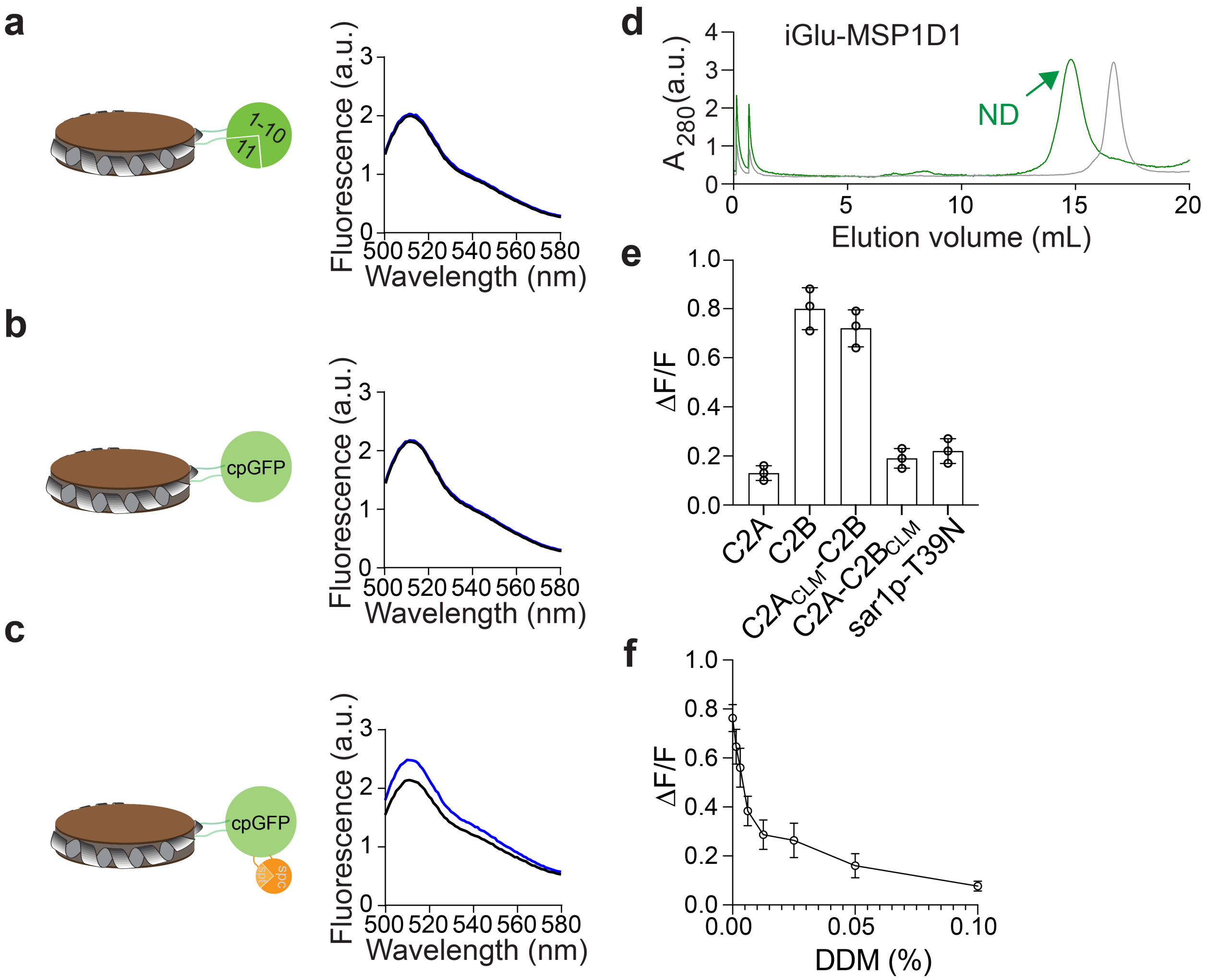


**Supplementary Figure 5. Optimization and characterization of isenND.**

(**a-c**) Illustration (left) and representative fluorescence spectrum (right) of the indicated nanodiscs before (black) and after (blue) the addition of syt1 in the presence of $Ca^{2+}$. A, $spGFP_{1-10/11}$-MSP1D1; B, spcpGFP-MSP1D1; C, SPY- spcpGFP-MSP1D1. (**d**) Representative SEC profiles of iGlu-MSP1D1 (gray) and nanodiscs (green). The protein: lipid ratios were 1:60 for these nanodisc reconstitutions. (**e**) Responses of the iGlu-MSP1D1 nanodisc to the indicated fragments and mutants of syt1 and sar1p. C2A and C2B are the tandem cytoplasmic C2 domains of syt1. CLM, $Ca^{2+}$ ligand mutations that disrupt the interaction of C2A and C2B with membranes. A previous study[1] showed that C2A and C2A-$C2B_{CLM}$ were defective in bending membrane, while C2B and $C2A_{CLM}$-C2B retained the membrane bending activity of the wild-type protein. (**f**) Responses of the iGlu-MSP1D1 nanodiscs to syt1-mediated membrane bending reaction in the presence of DDM at the indicated concentrations.

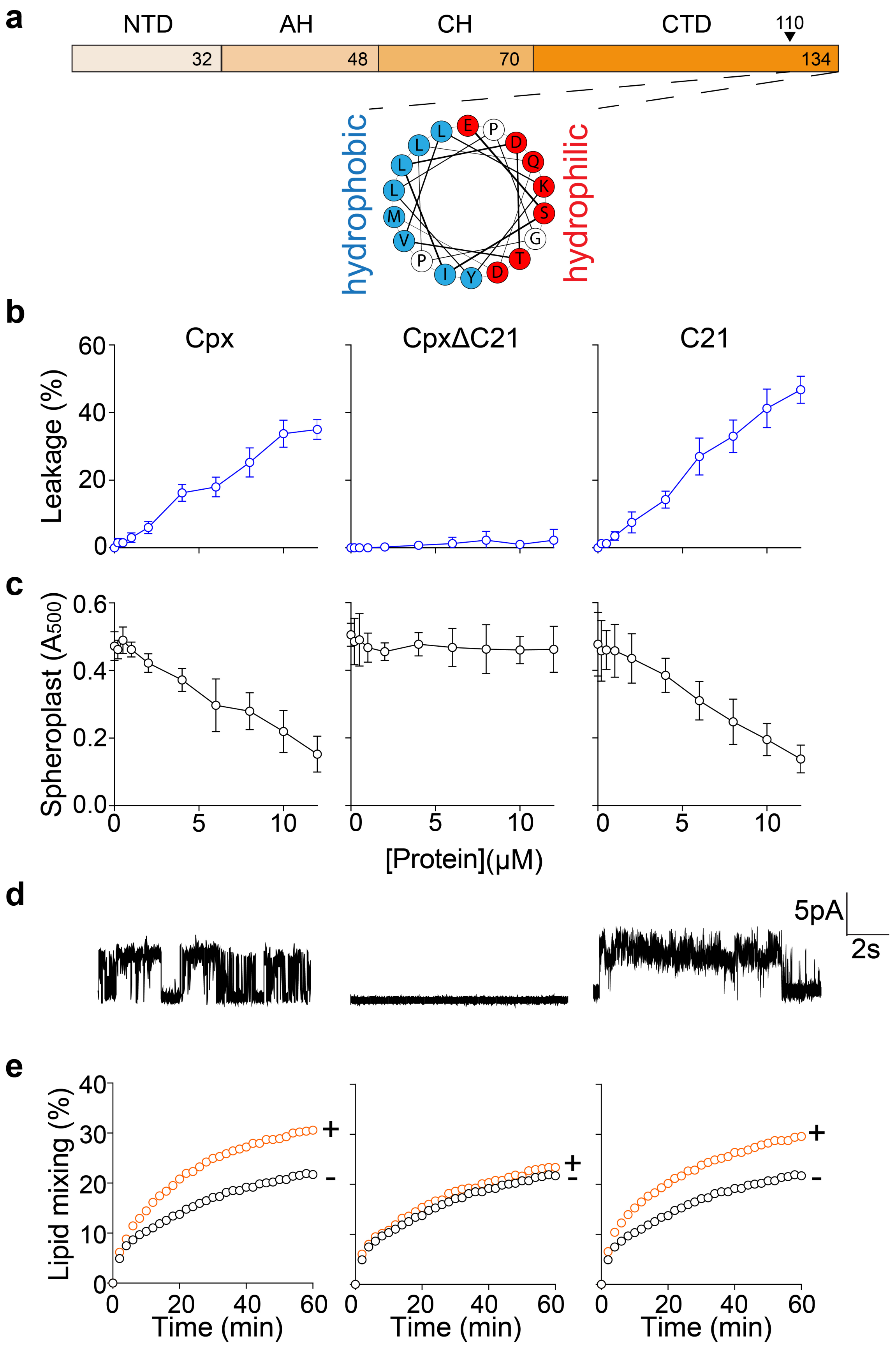

a
NTD
AH
CH
CTD
110
32
48
70
134
hydrophobic
hydrophilic
L
L
L
L
M
V
P
I
Y
D
T
G
S
K
Q
D
P
E
b
Cpx
CpxΔC21
C21
Leakage (%)
60
40
20
0
c
Spheroplast (A500)
0.6
0.4
0.2
0.0
0
5
10
[Protein](μM)
d
5pA
2s
e
Lipid mixing (%)
40
30
20
10
0
20
40
60
Time (min)
+
-

**Supplementary Figure 6. Complexin remodels membrane.**

(**a**) Top, diagram of mouse Cpx and the location of the C-terminal amphipathic helix. NTD, N-terminal domain; AH, accessory helix; CH, central helix; CTD, C-terminal domain. Bottom, Helical-wheel projection of the Cpx amphipathic helix (residues 114-134). (**b**) Leakage assays using liposomes loaded with glutamate. Upon addition of Cpx and C21, pore formation results in the efflux of glutamate, as monitored using the fluorescent sensor, iGluSnFR; CpxΔ114-134 was without effect. (**c**) Lysis of E. coli spheroplasts, monitored via absorbance at 500 nm, in response to Cpx and C21; again, CpxΔ114-134 was without effect. (**d**) Representative planar lipid bilayer recordings in the presence of Cpx, CpxΔ114-134, and C21; Cpx and C21 formed pores. (**e**) SNARE-mediated bilayer fusion assays included full-length syt1 and 0.5 mM $Ca^{2+}$, and were conducted in the absence (-) and presence (+) of Cpx, CpxΔ114-134 and C21. Data shown in panel b and c contained data published elsewhere[2] and additional replicates included in the statistical analysis.

**Supplementary References**


1. Hui, E.F., Johnson, C.P., Yao, J., Dunning, F.M. & Chapman, E.R. Synaptotagmin-Mediated Bending of the Target Membrane Is a Critical Step in $Ca^{2+}$-Regulated Fusion. *Cell* **138**, 709-721 (2009).
2. Courtney, K.C. et al. The complexin C-terminal amphipathic helix stabilizes the fusion pore open state by sculpting membranes. *Nat Struct Mol Biol* **29**, 97-107 (2022).
